# Typologies of Prescription Opioid Use in a Large Sample of Adults Assessed for Substance Abuse Treatment

**DOI:** 10.1371/journal.pone.0027244

**Published:** 2011-11-02

**Authors:** Traci C. Green, Ryan Black, Jill M. Grimes Serrano, Simon H. Budman, Stephen F. Butler

**Affiliations:** 1 Brown Medical School, Providence, Rhode Island, United States of America; 2 Rhode Island Hospital, Providence, Rhode Island, United States of America; 3 Inflexxion, Inc., Needham, Massachusetts, United States of America; Yale University School of Medicine, United States of America

## Abstract

**Background:**

As a population, non-medical prescription opioid users are not well-defined. We aimed to derive and describe typologies of prescription opioid use and nonmedical use using latent class analysis in an adult population being assessed for substance abuse treatment.

**Methods:**

Latent class analysis was applied to data from 26,314 unique respondents, aged 18-70, self-reporting past month use of a prescription opioid out of a total of 138,928 cases (18.9%) collected by the Addiction Severity Index-Multimedia Version (ASI-MV®), a national database for near real-time prescription opioid abuse surveillance. Data were obtained from November 2005 through December 2009. Substance abuse treatment, criminal justice, and public assistance programs in the United States submitted data to the ASI-MV database (n = 538). Six indicators of the latent classes derived from responses to the ASI-MV, a version of the ASI modified to collect prescription opioid abuse and chronic pain experience. The latent class analysis included respondent home ZIP code random effects to account for nesting of respondents within ZIP code.

**Results:**

A four-class adjusted latent class model fit best and defined clinically interpretable and relevant subgroups: Use as prescribed, Prescribed misusers, Medically healthy abusers, and Illicit users. Classes varied on key variables, including race/ethnicity, gender, concurrent substance abuse, duration of prescription opioid abuse, mental health problems, and ASI composite scores. Three of the four classes (81% of respondents) exhibited high potential risk for fatal opioid overdose; 18.4% exhibited risk factors for blood-borne infections.

**Conclusions:**

Multiple and distinct profiles of prescription opioid use were detected, suggesting a range of use typologies at differing risk for adverse events. Results may help clinicians and policy makers better focus overdose and blood-borne infection prevention efforts and intervention strategies for prescription opioid abuse reduction.

## Introduction

Since the 1960s, the availability and potency of prescription opioids in the United States (U.S.) has increased dramatically [1], [2], [3], with a concomitant increase in abuse of these medications [4]. Prescription opioids now outrank marijuana as the drug most associated with first time illicit drug use [4]. Non-medical use of prescription opioids is of public health concern because it is linked to serious personal health consequences, including addiction and fatal opioid overdose [5], injection drug use [6], and poly-drug use [7]. Drug-related deaths, primarily comprised of overdoses, overtook motor vehicle accidents in 2009 as the leading cause of accidental adult death in the US, an increase that has largely been attributed to greater involvement of prescription opioid medications [8], [9], [10]. Since 2002, deaths linked to prescription opioid medications outnumbered the total of deaths caused by heroin and cocaine combined [11]. A study by Hall and colleagues showed that 93.2% of all unintentional overdose deaths in West Virginia in 2006 could be attributed to prescription opioids [12].

As a population, non-medical prescription opioid users are not well-defined. Previous studies have focused on various demographic groups of non-medical prescription opioid users, ranging from young people [13], college students [14], [15], the elderly [16], women [17], [18], chronic pain patients [19], [20] [21], to street drug users [22]. These populations may differ drastically in important ways, including route(s) of administration of the prescription opioid, concurrent drug and alcohol use, treatment experience, and history of substance abuse dependence, among others. Such variability makes it difficult to summarize these disparate groups. Furthermore, clinicians and policy makers are faced with the challenge of focusing prevention, screening, intervention, and treatment strategies to address non-medical prescription opioid use in a way that prioritizes individuals at high risk of harm and poor health outcomes, without a clear sense of the nature of the disparate populations that may be involved.

The aim of this study was to derive and describe typologies of prescription opioid use in a large and diverse population of adults in treatment for substance abuse or dependence using latent class analysis. A latent class analysis approach assumes that the study population represents not one homogenous group of prescription opioid users but a mixture of several distinct subgroups of medical and non-medical prescription opioid users. These subgroups are latent, that is, they are not directly observable but they can be inferred based on similarities in individuals’ responses to questions about their health behaviors and non-medical prescription opioid use. Employing such an analysis, people are empirically divided into subgroups rather than categorized *a priori* or by study design. Latent variables are commonly applied in healthcare. For instance, the latent concept of “quality of life” refers to a measurement that cannot be directly observed but is instead derived from clinical observation and questions administered to a patient or caregiver.

## Methods

This study was approved by the Institutional Review Board of Rhode Island Hospital and determined to be exempt from the Federal Regulation 45 CFR 46. Data were de-identified for analysis; therefore, the Institutional Review Board of Rhode Island Hospital waived the need for consent.

### Sample

The study protocol sample consisted of respondents aged 18 to 70 years being assessed for substance abuse treatment at a treatment facility, criminal justice setting, or public assistance program across the U.S. who completed the Addiction Severity Index-Multimedia Version (ASI-MV®) (described below). At the time of this study, the ASI-MV database consisted of 138,928 assessments, collected from November 2005 through December 2009. These assessments, generally included as part of the intake process for substance abuse treatment, were conducted in 538 sites, serving patients from 474 unique resident 3-digit ZIP codes. Assessment sites in the ASI-MV network use the ASI-MV to for treatment planning and triage around substance abuse problems and associated life-functioning areas. Of the participating sites, 57% provide predominately substance abuse assessments for inpatient/residential treatment, outpatient non-methadone and methadone maintenance programs. Respondents also may have completed the ASI-MV as part of their experience in drug court, probation/parole, or Driving While Intoxicated (DWI) programs (approximately 33%) with another 7% classified as a combination of substance abuse and criminal justice assessments. Just over 2% of all assessments are conducted for TANF (Temporary Assistance for Needy Families) programs in order to determine need for treatment.

### Measures

#### ASI-MV

The ASI-MV is a proprietary data stream of the National Addictions Vigilance Intervention and Prevention Program (NAVIPPRO®) [23], a comprehensive risk management system for prescription opioids and other Schedule II and III therapeutic agents. The ASI-MV is a computer-administered version of the Addiction Severity Index (ASI); a standard intake assessment designed for use upon treatment admission with demonstrated reliability and validity [24]. The ASI-MV presents questions on the computer in both text and audio to address literacy limitations. In addition to the original ASI questions, the program collects product-specific, geographically-sensitive information about past 30 day prescription opioid use/abuse along with questions about route(s) of administration (oral, smoking, snorting, injecting), source of drug, whether they currently have a pain problem, and whether they have taken prescribed pain medication for their pain in the past 30 days. As in the original ASI [25], responses to the ASI-MV generate composite scores that reflect severity in seven problem areas known to be associated with substance use disorders: alcohol and drug use, employment and family/social functioning, and medical, legal, and psychiatric status. For each of these domain areas, objective questions measure the number, extent, and duration of symptoms in the past 30 days, along with subjective ratings of severity in each problem area. Items within each domain are mathematically combined to generate composite scores that range from 0 to 1 where higher scores reflect greater problem severity[25].

The software generates a psychosocial report and other documentation that is important clinically. As such, this assessment is part of the clinical flow and is not a separate survey or questionnaire [23]. Data from ASI-MV assessments are collected for clinical purposes. Once the patient completes the assessment, data are de-identified, made HIPAA compliant and uploaded to a central server. Network sites are not paid for these data which constitute a continuous, real-time data stream on substances used and abused by adult respondents (18 years or older) entering or being assessed for substance abuse treatment.

#### Latent class analysis Indicators

Six indicators were used to capture the latent variable of prescription opioid use. At the time of the analysis, use and non-medical use of 52 specific prescription opioid products were captured by the ASI-MV (Table 1). Two indicators of any past month non-medical use, one for short-acting and one for long-acting prescription opioid medications, were created based on responses to the product-specific items. In the ASI-MV, non-medical use is operationalized as self-reported past 30-day use of any prescription opioid “not in a way prescribed by your doctor, that is, for the way it makes you feel and not for pain relief.” A third indicator aimed to capture aspects of “intended route of administration”, taking into account the self-reported route of administration of the prescription opioid medication. Based on mounting evidence that a drug’s route of administration may signify different degrees of dependence or addiction [26], [27] and has public health implications for risk of blood borne virus transmission [28], [29], [30], [31], this indicator dichotomized use of a drug by any route of administration other than as intended (i.e., oral for most formulations). A fourth indicator reflected the source of the medication, dichotomized to a single, licit source (one’s own doctor) versus all other sources (e.g., dealer, friends/family). The final two indicators represented whether or not the respondent self-reported having either a chronic medical health problem or a pain problem and whether or not the respondent was taking a prescribed medication for a medical problem or receives help for a medical problem in the past 30 days. The dichotomized variables included as latent class indicators are listed in Table 2.

**Table 1:** Compounds and example prescription opioid medication products tracked by ASI-MV and included in analysis.

Compound	Example product: brand name or generic and manufacturer
Oxycodone extended release	OxyContin (before reformulation), oxycodone ER-Endo Pharmaceuticals
Oxycodone combination immediate release	Percocet, Tylox
Hydrocodone	Vicodin, Lortab
Meperidine	Demerol
Propoxyphene	Darvocet, Darvon
Fentanyl	Duragesic, Fentora
Oxymorphone	Opana ER, Opana IR
Hydromorphone	Dilaudid, Palladone
Codeine	Tylenol with codeine
Morphine	MS Contin, KADIAN
Tramadol	Utram, Ultracet
Methadone*	methadone—Covidien Pharmaceuticals, methadone—Roxane Laboratories
Buprenorphine	Subutex, Suboxone
Pentazocine	Talwin
Butorphanol	Stadol

*Includes methadone products used in the treatment of chronic pain only.

ER = extended release, IR = immediate release.

Note: This is a partial listing of the brand name and generic opioid analgesics tracked by ASI-MV and used in this analysis. Please contact Inflexxion, Inc. for the full list of products included in this analysis or for information on all Schedule II and III medications tracked by the ASI-MV.

**Table 2:** Class prevalence among the study sample and adjusted probabilities of responding to each indicator conditional upon membership in the latent class.

	Class 1Use as prescribed*N = 4,973	Class 2Prescribed misusersN = 7,079	Class 3Medically healthy abusersN = 9,420	Class 4Illicit usersN = 4,842
Class Prevalence	18.9%	26.9%	35.8%	18.4%
Indicators: ‘Yes’ response to the following				
Nonmedical use of Short acting prescription opioid	0.0761	0.7545	0.7512	0.8161
Nonmedical use of Long acting prescription opioid	0.0031	0.4682	0.5091	0.9236
Use by non-medical route of administration	0.0111	0.2430	0.3374	0.9089
Illicit source (i.e., not one’s own, single physician)	0.0005	0.4773	0.8816	0.9994
Has a current chronic medical healthproblem/pain problem	1.00	0.9706	0.5138	0.4346
Takes prescribed medication for a medical problem/Receives help for a medical problem, past 30 days	0.9485	0.8863	0.6068	0.4859

*To understand this table’s content, take for instance class 1, which had a prevalence of 18.9%. Conditional upon membership in this class, class 1 respondents had very low adjusted probabilities (close to 0) of responding ‘Yes’ to the first 4 indicators listed and very high adjusted probabilities (close to 1.0) of responding ‘Yes’ to the last two indicators: ‘Has a current chronic medical health problem/pain problem’ and ‘Takes prescribed medication for a medical problem/Receives help for a medical problem, past 30 days’. Based on this pattern of response, class 1 was labeled, for ease of discussion, as ‘use as prescribed’.

#### Covariates

Adjusted latent class analysis models considered three demographic covariates: age, minority status (White, non-White), and sex. Cross-class comparisons were conducted to compare other sociodemographics from the ASI-MV, including employment and educational status, living situation, incarceration history, U.S. Census region of respondent’s residence, history of abuse (physical, sexual), self-reported concurrent medical and psychiatric co-morbidities, current prescription for psychiatric medication, current use of alcohol to intoxication (i.e., at least five drinks in a day for men, four for women), duration of illicit drug use by substance, overdose history, and recent (past year) initiation of heroin and injection drug use. Finally, ASI composite scores were examined. An ASI composite score [25], [32] was calculated for each problem domain and represents current (past 30-day) problem severity of the respective domain.

### Statistical analysis

We used latent class analysis, a statistical method for discovering subgroups, or latent classes, in a population. The latent class analysis results in estimates of: (a) the prevalence of each latent class (i.e., prior probability that a randomly chosen person will be in each class), and (b) the probability of response to an indicator, conditional on the latent class. Class categorizations are based on the prevalence of the latent classes and are mutually exclusive and exhaustive; once an individual is determined to belong to a certain latent class they cannot be a member of another latent class. Latent class analysis can also incorporate covariates which may influence class membership [33]; multi-level latent class analysis can incorporate clustering of responses, for instance, at the geographic level, as random effects [34]. A random effect for the respondent 3-digit ZIP code was included in the model. Latent class analysis methods have been used in several recent studies on drug and alcohol abuse [35], [36].

To identify the optimal number of latent classes and the best fitting model, we sought to minimize the Bayesian Information Criterion (BIC) —a goodness-of-fit measure used in model selection that takes into account the number of parameters in the model—and yield interpretable latent classes of >1% prevalence (i.e., avoiding obscure, unstable class sizes). The BIC was selected over other goodness-of-fit criteria (e.g., Akaike’s Information Criterion) for latent class analysis based on its performance in simulation studies [37]. Unadjusted models considered one to eight classes. Eight was chosen as the upper limit of the possible number of latent classes, to leave open the opportunity for identifying unique trends. However, a balance between parsimony, meaningful differences in the addition of more numerous classes and, ultimately, fulfillment of the BIC and prevalence criteria took precedent. Once a final model was selected, first we incorporated the random effect for ZIP code, and then we included the covariates in a stepped fashion individually and summarily in adjusted latent class analysis models [38]. The modal class was used for cross-class comparisons. Mplus version 5.2 [34] and Latent Gold version 4.5 software were used to fit the latent class analysis models; all class comparisons were conducted in SAS v.9.2.

To better characterize the adjusted latent class analysis results, we tabulated class-specific descriptive statistics for the socio-demographic and substance abuse covariates, and conducted Pearson χ^2^ tests of categorical variables and ANOVAs with Tukey-Kramer post-hoc tests for multiple pairwise comparisons of the ASI composite scores. In addition, to provide evidence that the classes differed in clinically meaningful and public health-relevant ways, we evaluated the risk of fatal and nonfatal opioid overdose and of blood-borne infection for each class. A high level of risk of fatal and nonfatal opioid overdose was based on the prevalence of risk factors known to be associated with fatal and nonfatal overdose (i.e., heavy alcohol use, benzodiazepine/sedative use, use by injection, poly-opioid use, incarceration history, comorbid medical conditions) [39], [40], [41], [42]. High risk of blood-borne infection was assigned based on drug use by injection [28], [43], [44]. Elevated risk was assigned based on non-injection drug use associated with transmission of HIV, namely crack/cocaine use; low risk was assigned based on absence or low prevalence of risk factors associated with high or elevated risk.

## Results

### Demographics of ASI-MV Population and latent class analysis study sample

The mean age of the full sample (N = 138,928) was 34.5 years (S.D. = 11.6 years); 64.1% of the sample was male and 53% was non-Hispanic White, with 16% African American and 24.2% Hispanic/Latino. Of the sample, 30.5% reported having chronic medical problems and 31.0% reported having a pain problem (i.e., "Do you have a pain problem? That is, a physical pain that is more than the usual aches and pains?"). Approximately nineteen percent (18.9%) of respondents reported use of prescription opioids in the past 30 days and were aged 18 to 70 years. These 26,314 unique respondents served as the latent class analysis study sample.

The mean age for the latent class analysis sample (N = 26,314) was 35.2 years (S.D. 11.3 years); 56.4% were male and 63.6% were of non-Hispanic White race/ethnicity, with 9.9% African American, 22.0% Hispanic/Latino, and 5.0% of other race/ethnicity. Among these respondents, 53.3% reported having chronic medical problems and 66.4% reported having a pain problem.

### Determination of the number of latent classes

A four-class unadjusted model fit best: the prevalence of each class exceeded 5% and, while no minimum BIC was reached at this point in the modeling, the difference in BICs was minimal (BIC 5 class  = 159042 vs. BIC 4 class  = 159129). The four-class model defined clinically interpretable and relevant subgroups, labeled based on their pattern of item-response probabilities and for discussion purposes as: Use as prescribed, Prescribed misusers, Medically healthy abusers, and Illicit users. More specifically, Table 2 reports the probability of responding ‘yes’ to each of the six self-reported latent class indicator items (Table 2), conditional upon membership in the given latent prescription opioid use class, and the relative prevalence of each class. Model fit improved after adjusting for age, minority status, and sex (BIC  = 154341). All bivariate residuals among the six latent class indicators were high, and as a result, direct effects were incorporated into the model to account for these residual correlations. Three high bivariate residual values between latent class indicators and the covariates were also detected; inclusion of these direct effects further improved the model fit (BIC  = 154099).

#### Class characteristics and cross-class comparisons

Table 3 presents class characteristics and differences across key socio-demographic and substance abuse covariates. All calculated differences in the cross-class and pairwise comparisons were statistically significant (p<.05).

**Table 3:** Socio-demographic and substance abuse covariates (%) of the four latent prescription opioid use classes (N = 26,314).

Suggested Name	Class 1 Use as prescribed	Class 2 Prescribed misusers	Class 3 Medically healthy abusers	Class 4 Illicit users
**Mean age (SD)**	40.2 (10.9)	42.6 (10.2)	31.3 (9.1)	26.9 (6.9)
**Non-Hispanic White race**	60.1	51.5	61.4	86.7
**Female**	45.2	50.3	39.6	39.5
**Married**	27.9	27.2	21.1	15.6
**Less than high school education**	29.0	30.3	31.4	28.0
**Usual full/part-time employment, past 3 years**	38.8	35.4	54.8	53.9
**Employment problems >50% of days paid in past 30**	37.4	41.1	48.0	50.4
**Past year incarceration**	16.6	16.3	21.6	20.3
**Concurrent substance abuse**				
Cocaine/crack	9.4	17.9	25.6	37.5
Amphetamine	3.3	7.6	12.6	20.5
Sedative	21.7	29.3	30.7	46.7
Methadone	7.3	15.6	15.9	33.5
Alcohol to intoxication>3 days/wk	5.6	11.3	15.9	15.7
**Heroin history**				
No heroin use	90.8	78.1	75.5	63.2
Heroin use ≥1 year, not current user	6.2	10.2	7.0	5.8
Current heroin use, not new initiate	1.8	8.1	11.3	15.7
Past year initiate to heroin use	1.2	3.6	6.2	15.2
**Injection history**				
Never injected	82.0	67.4	66.9	47.9
Ever injected, not new initiate	16.6	27.4	25.1	31.8
New initiate to injection	1.4	5.1	8.0	20.4
**Past year initiation of non-medical prescription opioid use**				
Initiated with 1 prescription opioid	0.5	8.8	11.7	2.4
Initiated with >1 prescription opioid	0	6.7	11.2	17.1
**Duration of use** *				
Illicit drugs				
0 years	61.3	48.9	41.1	31.4
More than 3 years	19.0	27.4	26.4	23.2
Non-medical use of therapeutics				
0 years	67.1	48.7	50.0	27.4
More than 3 years	14.6	23.9	15.7	23.5
**Primary problem**				
Heroin	5.3	12.9	15.4	18.2
Prescription opioids	5.1	13.6	17.0	41.4
**Mental health**				
History of depression	72.6	80.1	74.1	73.9
History of anxiety	73.8	80.3	73.5	74.6
Past suicide attempts or ideation	6.1	11.3	9.8	10.2
History of physical abuse	51.5	57.9	48.8	44.2
History of sexual abuse	30.2	36.3	27.6	25.0
Prescribed psychiatric medications	40.1	42.7	26.9	26.1

All variables are statistically significant (p<0.05 or less) from one another, based on Pearson χ^2^ tests of categorical variables (df = 3, χ^2^ values larger than critical value 7.81) and ANOVA (F(3, 26,311) =  3,057, (p<0.001)) with Tukey-Kramer post-hoc tests for multiple pairwise comparisons for the age variable.

SD = standard deviation.

* For duration of use, illicit drugs include cocaine, amphetamines, hallucinogens, and inhalants; heroin is presented separately. Therapeutics include benzodiazepines, antidepressants, and methadone and exclude all other prescription opioids.

Respondents in the Use as prescribed class (class 1) and Prescribed misusers class (class 2) were the oldest, and the Illicit users (class 4) the youngest. The Use as prescribed class was characterized by its older age, medical use, lack of recent employment, and general lack of problematic drug use, including non-medical prescription opioid use. In contrast, the Prescribed misusers class (class 2) exhibited similar medical problems to those using as prescribed, had proportionately more females, greater racial diversity, histories of current and past drug abuse, and, uniquely, self-reported the highest rates of sexual and physical abuse histories, lifetime depression, lifetime anxiety, and currently prescribed psychiatric medications of all the classes. The Medically healthy abusers class (class 3) was distinct from the first two classes in its younger age demographic, lower education levels, and extensive history of heavy drinking and illicit drug use. They also reported using alcohol to intoxication the most often. Both the Medically healthy abusers and Illicit users (class 4) classes exhibited greater criminal involvement, recent employment problems, current illicit drug use, and recent initiation of non-medical use of prescription opioids, heroin, and injection drug use. The Medically healthy abusers class reported similar, sizeable proportions of past year initiation of nonmedical use of one and multiple prescription opioid medications. The Illicit users class were more likely to report recent initiation of multiple rather than one prescription opioid. Though they were the youngest of the four groups, the Illicit users reported a long history of abuse of therapeutic drugs and, more recently, illicit drug use, including initiation of heroin and other drug use by injection. Prescription opioids were indicated as the primary problem drug by 2 of every 5 people in the Illicit users class. Demographically, the Illicit users class were comprised of mostly non-Hispanic Whites (86.7%), males (60.5%), and had the lowest prevalence of marriage of the classes.

#### Class differences by ASI Composite scores

Table 4 displays the between-class differences for the composite scores of each of the seven ASI domains. All comparisons returned statistically significant cross-class comparisons, except where noted in the table. Class 1 (Use as prescribed) had the highest ASI composite scores in the medical domain. Class 2 (Prescribed misusers) also scored high on the medical domain and exhibited the highest psychiatric domain score of all classes. Class 3 (Medically healthy abusers) showed the highest composite score in the alcohol domain but low scores in the medical domain. The highest composite scores in the drug and legal ASI domains were detected among members of class 4 (Illicit users).

**Table 4:** Addiction Severity Index composite scores by latent prescription opioid use class.

Suggested Name	Class 1Use as prescribed	Class 2Prescribed misusers	Class 3Medically healthy abusers	Class 4Illicit users
Class Prevalence	18.9%	26.9%	35.8%	18.4%
**Addiction Severity Index Composite Scores, Mean (SD)**				
Alcohol	0.11 (0.19)	0.17 (0.25)^a^	0.21 (0.26)	0.19 (0.25)^a^
Drug	0.09 (0.10)	0.16 (0.13)	0.19 (0.14)	0.31 (0.13)
Employment	0.65 (0.30)^a^	0.67 (0.30)^a^	0.63 (0.31)^ b^	0.62 (0.30)^ b^
Family	0.20 (0.20)	0.26 (0.22)^a^	0.27 (0.21)^a^	0.30 (0.21)
Medical	0.70 (0.24)^ a^	0.69 (0.26)^ a^	0.37 (0.32)	0.36 (0.32)
Legal	0.14 (0.18)^a^	0.16 (0.20)^ a^	0.20 (0.21)	0.24 (0.24)
Psychiatric	0.32 (0.26)	0.40 (0.27)	0.34 (0.26)^ b^	0.35 (0.25)^ b^

All one-way ANOVAs were statistically significant (p<0.05 or less) from one another, conducted with F (3, 26,311). The one-way ANOVAs returned results larger than the critical value of 2.70 (p = 0.05) or 3.98 (p = 0.01). In post-hoc comparisons of the ASI composites across classes, same letter superscripts denote statistically similar values, where p≥.05 in Tukey-Kramer post-hoc ANOVA tests. All other post-hoc comparisons were statistically different from one another. For instance, for ASI Employment, classes 1 and 2 have similar values (p>0.05) which are statistically different from classes 3 and 4 (p<0.05).

SD = standard deviation.

#### Potential risk of overdose and of blood-borne viral infection

Three of the latent class analysis classes evidenced high potential for fatal and nonfatal opioid overdose (Table 5): Prescribed misusers, Medically healthy abusers, and Illicit users, based on their co-prescribed psychiatric medications, problem drinking, recent incarceration, and concurrent opioid use, often by injection. The highest potential risk of blood-borne viral infection was found among the Illicit users (class 4), with Medically healthy abusers at elevated risk of infection.

**Table 5:** Overdose and blood-borne viral infection risk potential of the four latent prescription opioid use classes.

Fatal and nonfatal opioid overdose				
*Risk factor*	Class 1Use as prescribed	Class 2Prescribed misusers	Class 3Medically healthy abusers	Class 4Illicit users
Change in toleranceIncarceration history			X	X
IllnessComorbid medical conditions/highest ASI medical composite score	X	X		
Use drugs alone*Not assessed*				
Mixing/poly-pharmacyHeavy alcohol use/highest ASI alcohol composite score			X	
>30% prevalence sedative use/highest ASI psychiatric score		X	X	X
Poly-opioid use				X
Dose/routeHistory or recent initiation of drug use by injection		X	X	X
**Overall overdose risk potential**	**Elevated**	**High**	**High**	**High**

Blood-borne viral infection (HCV, HIV, HBV)				
*Risk factor*	Class 1Use as prescribed	Class 2Prescribed misusers	Class 3Medically healthy abusers	Class 4Illicit users
Recent initiation of injection				X
Crack/cocaine use			X	X
**Overall blood-borne viral infection potential risk**	**Low**	**Low**	**Elevated**	**High**

‘X’ indicates risk factor present at 20% or greater and/or highest related ASI composite score in class; Low risk potential = no risk factors present; Elevated risk potential = one risk factor present; High risk potential = two or more risk factors present.

HCV = hepatitis C virus, HBV = hepatitis B virus, HIV = human immunodeficiency virus.

## Discussion

The current study is the first latent class analysis of prescription opioid use in a population of individuals being assessed for substance use problems from a large and diverse sample of such respondents in the U.S. Due to the high degree of specificity of the ASI-MV database, we were able to explore patterns of prescription opioid use that incorporated route of administration, source of drug, and product-level indicators of non-medical use for short and long-acting opioid medications. Our results show that four unique groups of prescription opioid users could be identified within this sample: use as prescribed class (class 1), prescribed misusers class (class 2), medically healthy abusers class (class 3) and illicit users (class 4). These groups differed in key ways relevant to public health and clinical intervention, including: age, race/ethnicity, concurrent drug use, onset and duration of their drug use, routes of administration, and comorbid psychiatric and medical problems, among others.

Several of our findings converge with other studies of trends in prescription opioid abuse. Data comparing 1998 to 2008 substance abuse treatment admissions involving prescription opioid pain relievers from the Treatment Episode Data Set (TEDS) showed a four-fold increase in admissions, with notable increases in the proportion of people reporting pain reliever abuse with a co-occurring psychiatric disorder [45]. In general, the TEDS sample of prescription opioid abusers is demographically similar to the current study population (i.e., predominantly non-Hispanic White, aged 18-34 years, sizeable and growing proportion of females). Two published latent class analyses have addressed non-medical use of prescription opioids in the U.S., using random national household samples. The first study, analyzing data from the 2002–2003 National Survey on Drug Use and Health (NSDUH), also identified a four class model of opioid analgesic users based only on measures of dependence [46]. In this study, a class of users was described with low probability of endorsing any of the seven symptoms of dependence and another class of users with high probability of endorsing each of the seven symptoms of dependence, potentially similar to our classes 1 and 4, respectively. Two other classes were characterized as more moderate in terms of endorsing symptoms of dependence. Wu et al. also conducted a latent class analysis, using data from respondents to the National Epidemiologic Survey on Alcohol and Related Conditions (NESARC), but their analysis considered those who self-reported non-medical prescription opioid use as defined by the survey tool [47]. The authors discovered four subtypes of non-medical prescription opioid users, linking several types to high rates of major depression and disability in the mental health domains and with differences by gender in patterns of other drug use combined with non-medical prescription opioid use. However, the NSDUH and NESARC are household-based samples and exclude incarcerated populations, those who are homeless, and other marginalized populations whose exclusion or non-participation may lead to an under-counting of the extent and nature of drug use in the community.

McCabe et al. characterized college-aged non-medical users of prescription opioids *a priori* into subgroups based on motive, route of administration, and co-ingestion with alcohol [48]. Interestingly, similar to the Class 2 (Prescribed misusers) discovered in the current analysis, one group in the McCabe et al. study was described as “self-treatment types”, who used mainly oral routes of administration, had no co-ingestion with alcohol, and were motivated by wanting to achieve greater pain relief. Also described was a “recreation subtype” group, which appeared qualitatively similar to those in our Class 3 (Medically healthy abusers) in that they reported co-ingestion with alcohol. Different from these prior studies, our approach employed as latent class indicators product-specific route of administration and drug source questions collected by the ASI-MV, and it considered as the population at risk for non-medical prescription opioid use all persons currently using these medications. Lacking information on motivations for use, this expanded risk pool was crucial to exploring how medication prescribed to an individual is potentially being used or misused. In addition, our analyses and data interpretation take a public health approach, aiming to highlight not just the clinical salience of these subgroups to treatment providers but also the association of the discovered subpopulations with important drug use consequences (i.e., overdose, blood borne virus) of public health import. These results represent a unique contribution to the descriptive epidemiology of prescription opioid use in the U.S.

Prescription opioid abuse is a well-characterized phenomenon among pain patients [23], [49], [50], [51]. A recent study [52] reported that 47% of persons presenting for treatment from oxycodone addiction had their first exposure to opioids through a prescription for pain relief and that 31% had no prior history of substance abuse. However, a recent systematic review found that patients with chronic non-cancer pain who had comorbid substance use disorders are more likely to be prescribed opioids and higher doses of opioid medications compared with patients who do not have a history of substance use disorders despite similar pain outcomes [53]. While our data do not permit us to determine whether the Prescribed misusers class represent people undiagnosed or under-treated for physical or psychic pain or some other underlying medical condition, our findings indicate that this class is in contact with the medical community, has obtained access to prescribed medications, and reports misusing them. It is crucial to recognize that this group represents approximately a quarter of our sample; most people reporting non-medical prescription opioid use are not patients misusing their medications. Calls to restrict people with a substance abuse history from receiving prescription opioid medications for pain appear unjustified.

The class referred to as Medically healthy abusers (Class 3) account for the largest group in this analysis (35.8%). Associations between past-year non-medical use of prescription opioids and alcohol have been reported in other studies, including results from the general, non-treatment seeking population [54], [55], [56] and among college students [57], [58], [59], [60], [61]. Indeed, other studies have shown that those who report misuse of prescription opioids for pain relief (possibly analogous to Class 2 in the current latent class analysis), report less co-ingestion with alcohol than those who are misusing for reasons other than pain relief, such as those in Class 3 [48], [62]. Given the higher alcohol problems in this and other classes, prevention efforts could consider screening for prescription opioid abuse in driving while intoxicated cases and detoxification and outpatient treatment programs for patients with alcohol abuse and dependence.

Illicit users (class 4) scored the highest on the Drug and Legal domains of the ASI (indicating greater problems), reported a high prevalence of recent initiation of non-medical use of multiple prescription opioid medications in the past year, and had high prevalence of ever and recent initiation of injecting. Similar socio-demographic and concurrent drug use characteristics have been observed among street drug users in New York City [63] and among rural drug users located in Kentucky [64] and Ohio [65], especially in regards to crack cocaine use. In their qualitative study of prescription opioid users in NYC, Davis et al. conceptually categorized the population of prescription drug users into five subgroups, including an “illicit ingestion” group [22]. Several characteristics displayed by the Illicit users class (i.e., injection drug use) put them at increased risk for dependence [66]. Other authors have detected populations of non-medical prescription opioid users who report injection [67], [68], [69], often manifesting in areas of the South [69], [70]. Snorting of prescription opioids has also been described in young people [15] and is associated with experiencing substance use-related problems. Taken together, it appears that this class moves quickly to opioid addiction and is at extremely high risk of more severe addiction, opioid-related morbidity, and potentially, death.

**Figure 1:**
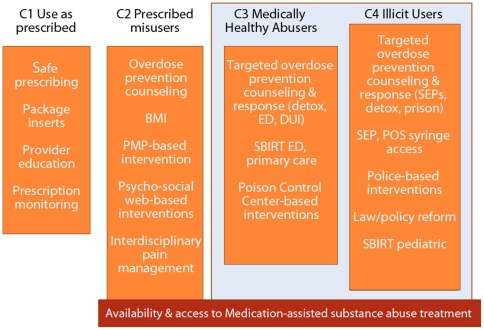
Latent class-specific targeted interventions to reduce risk of addiction, overdose, and transmission of blood-borne viruses. C1-C4 refer to latent class 1 to 4. Classes within the shaded area represent those subpopulations less likely to be reached through medical care providers alone. BMI = brief motivational interviewing; PMP = prescription monitoring program; ED = emergency department; DUI = driving under the influence; SBIRT = screen, brief intervention, referral to treatment approach; SEP = syringe exchange program; POS = point of sale (e.g., pharmacy).

Results further suggest the risk of serious health consequences borne by many of the prescription opioid use classes. Due to their frequent use of alcohol to intoxication, concurrent use of benzodiazepines, drug use by injection, history of incarceration, high prevalence of comorbid conditions, and/or poly-opioid use behaviors [39], [40], [42], [71], up to 81% of people in this sample (i.e., sum of prevalence for classes 2–4) are at high risk of fatal and nonfatal opioid overdose (Table 5). Risk of blood-borne viruses such as hepatitis B and C and HIV, transmitted most efficiently by injection [28], [29], [30], [31], could be high for up to 18.4% (i.e., prevalence for class 4: Illicit users) of the current study population (Table 5). Sero-prevalence studies could be conducted to verify differential prevalence and risk by prescription opioid use typology. Our results may be useful in posing these and additional research questions and, as suggested in Figure 1, in formulating better targeted preventive interventions to reduce fatal and nonfatal opioid overdose and blood-borne infection in the community.

Our results add to a growing body of research indicating that prescription opioid products are being misused and abused in ways that call for more nuanced and public health-oriented post-marketing surveillance and risk management responses than have been proposed heretofore. The main thrusts of the current Prescription Opioid Abuse National Strategy [72] and the Food and Drug Administration’s Risk Evaluation and Mitigation Strategy components [73] rely heavily upon provider and patient education and expansion of prescription monitoring programs. Based on our findings, such efforts will have limited effect on the largest subpopulations of non-medical prescription opioid users who are at greatest risk of adverse health events. These subpopulations are sizeable, exist primarily outside of the regular care of medical professionals, and would likely require other, very different intervention techniques, as Figure 1 suggests, such as targeted overdose prevention counseling and response [74], [75], [76] and health or social service venue-based preventive interventions (e.g., syringe exchange/delivery programs, programs tailored to drug detoxification program attendees), to reduce their risk of addiction, transmission of blood-borne viruses, and overdose. A comprehensive public health approach that incorporates supply and demand reduction, adequately extends and supports harm reduction and treatment, and recognizes the need for effective intervention at the individual and structural levels is indicated.

Strengths of the present study include the large and geographically diverse sample that includes both males and females of varying ages. Detailed data were obtained through the ASI-MV which allowed us to detect and describe unique classes based on a variety of factors important to issues related to prescription opioid use, such as the route of administration, presence of pain/medical problems, and other substances concurrently abused as well as to incorporate a random effect to account for correlations at the patient ZIP code level. Also, similarities between the discovered typologies, governmental statistics (e.g., TEDS), and previous studies conducted among select populations provide a degree of construct validity to the latent class analysis findings.

Limitations of this study include the use of cross-sectional data. To determine whether the identified groups are unique or represent stages in a progression of non-medical prescription opioid use, a longitudinal study would be needed. Another limitation is the reliance on self-reported data on substance use from a sample of people abusing drugs and alcohol. Though research and reviews continue to support the reliability and validity of self-report of patients entering substance abuse treatment [77], [78], [79], [80], [81], [82], measurement error, in the form of reporting biases, may be present in the ASI-MV data but would be expected to be non-differential, when present, leading to under-estimates of observed associations. True associations and prevalences may be larger. A third limitation is that the sample includes prescription opioid users currently being evaluated in substance abuse treatment settings, the criminal justice system, and/or receiving public assistance, and therefore may not be reflective of all prescription opioid users. The substance abuse and criminal justice focus of most sites may capture important avenues through which individuals who engage in prescription opioid abuse enter a treatment system. However, the findings of this study may not be directly comparable to other substance abuse treatment datasets, such as the Treatment Episode Data Set [83]. The ASI-MV sample does not capture data from children and adolescents under the age of 18, a population with potentially differing patterns of non-medical prescription opioid use, so our results may not generalize to them. It is also important to point out that the ASI-MV network provides a sentinel surveillance sample, useful for detecting trends and patterns in drug use. However, estimates may be limited in generalizability across places and populations, especially those with low participation in the ASI-MV network. As the purpose of this analysis was not to generate population estimates but rather to uncover patterns and trends in prescription opioid use, the large, product-specific and geographically diverse sample were key. Unmeasured covariates that would have been of interest to the present analysis include smoking status, duration of use of specific prescription opioid products, motivations for use of prescription opioids, and indicators of abuse or dependence criteria. Last, it would be important to replicate the latent class analysis findings in another, large sample of prescription opioid users, to test the predictive validity of the identified classes, and to explore their utility in tailoring pharmacovigilance, prevention and intervention efforts.

In conclusion, this study detected multiple and distinct profiles of prescription opioid users, suggesting a range of typologies rather than a simple dichotomy of those who do or do not report non-medical use of prescription opioids. For most patterns, non-medical prescription opioid use did not occur in isolation of abuse of other substances. The prominence of comorbid psychiatric and medical problems suggest the need for better integration of and access to mental health, primary care and substance abuse treatment.
